# Gender equity in the scientific workforce: what is the current state of pediatrics?

**DOI:** 10.1038/s41390-022-02235-0

**Published:** 2022-09-06

**Authors:** Elena Fuentes-Afflick, Stephen Daniels, Nancy D. Spector, Stephanie D. Davis, Tamara D. Simon

**Affiliations:** 1 Department of Pediatrics, Zuckerberg San Francisco General Hospital and the University of California, San Francisco, CA USA; 2 Department of Pediatrics, University of Colorado School of Medicine, Children’s Hospital Colorado, Aurora, CO USA; 3 Department of Pediatrics and Executive Leadership in Academic Medicine® (ELAM) Program, Drexel University College of Medicine, Philadelphia, PA USA; 4 Department of Pediatrics, University of North Carolina at Chapel Hill School of Medicine, UNC Children’s, Chapel Hill, NC USA; 5 Department of Pediatrics, Keck School of Medicine at the University of Southern California, Children’s Hospital Los Angeles, Los Angeles, CA USA

This executive summary represents a review of a SPR (Society of Pediatric Research) webinar entitled *Gender Equity in the Scientific Workforce: What Is the Current Status of Pediatrics?*^1^ The entire transcribed webinar is located on the SPR website.^2^

Over the past 30 years, we have experienced a major influx of women into the field of pediatrics. The distribution of female physicians varies by specialty and pediatrics is at the forefront of the gender transformation with 63% of pediatricians being women.^3^

Pediatrics should be leading the way in medicine to advance women in leadership. Women in academic pediatrics constitute 57% of the faculty and 26% of the chairs; women physicians account for 17% of deans at medical schools.^4^ In 2015, a *JAMA Pediatrics* article reported that 1 of 13 women medical school deans was a pediatrician.^5^ A 2019 *New England Journal of Medicine* article noted that without term limits in academic medicine, gender parity for deans of medical schools will not occur until the year 2070.^6^

As part of this webinar, three experts provide a blueprint of recommendations for faculty and leaders within academic medical centers to promote equity in the pediatric scientific workforce.

- *Stop blaming women; “the time is now” to start moving and changing systems*.
- *Search committees need to encourage candidates from diverse backgrounds to apply. Look at every search as an opportunity to improve diversity in the department. All search committee members should have specific training in implicit bias*.
- *To mitigate any kind of bias in the text of the job description, the search committee needs to review the job description text through the lens of different perspectives*.
- *Carefully review recommendation letters that have been written and consider whether they contain gendered terms*.
- *Gender-based differences in compensation are common; institutional salary equity analysis is important*.
- *Innovative educational models are needed to promote a diverse work environment*.
- *Leaders should assure that strategies are available to support and retain faculty on academic tracks when they have family responsibilities*.
- *People need a range of mentors to be able to help navigate the complex environment in academic medicine. Women faculty and faculty from diverse backgrounds must have leadership development opportunities for career advancement*.
- *Women are more likely to have mentors, and more likely to have multiple mentors, but less likely to get promoted. This is partly due to lack of sponsorship. A sponsor provides the “inside scoop” or background to help lead to one’s success. Be proactive about sponsorship*.
- *Partner with others to develop an approach to support male allyship*.
- *The goal for leadership teams, boards, and committees is to at least assure that 30% are women. This will ensure change in structure and culture. That’s because when women or people of underrepresented groups or those who experience intersectionality are in leadership positions, there is more visibility. California is paving the way by publishing standards of how many women should be on boards*.
- *Promote term limits for leadership positions and committee roles*.
- *Encourage conference planners to promote diversity through the inclusion of women and underrepresented people. Avoid “manels”*.
- *Nominate women for awards*.
- *Be transparent and share data related to equity, diversity and inclusion. Pick meaningful metrics to follow. Investigate causality, implement strategic interventions, track outcomes and adjust strategies, and then publish and disseminate the results*.
- *Research is needed to further study and understand the impact of gender and other demographic transitions for our field*.
- *Unless we collaborate, share resources, and move together, we will not be successful.*
